# From crisis to care: evaluating nursing competencies in pediatric intensive care units through family eyes

**DOI:** 10.1186/s12912-026-04850-2

**Published:** 2026-06-16

**Authors:** Eman Arafa Badr, Heba Hashem Monged, Sally Mostafa Mohamed

**Affiliations:** 1 Pediatric Nursing Department, Faculty of Nursing, Alexandria University, Alexandria, Egypt; 2 Critical Care and Emergency Nursing Department, Faculty of Nursing, Alexandria University, Alexandria, Egypt; 3 Pediatric Nursing Department, Faculty of Nursing, Rashid University, Rashid, Egypt

**Keywords:** Family satisfaction, Nursing care competencies, Critically Ill children, Pediatric intensive care unit

## Abstract

**Background:**

Pediatric nurses play pillar roles in providing continuous, holistic care for critically ill children. Hence, family satisfaction and nursing care competencies have surfaced as critical channels of quality of care in pediatric intensive care units.

**Aim:**

This study aimed to investigate the relationship between children’s family satisfaction and nursing care competencies in Pediatric Intensive Care Units.

**Methods:**

A cross-sectional correlational research design was conducted in five pediatric intensive care units at children’s hospitals related to different health sectors in Alexandria. A convenient sample of 150 pediatric critical care nurses who provided direct care for critically ill children in Pediatric Intensive Care Units (PICUs) and 218 families of critically ill children. The children’s socio-demographic and clinical data record, the pediatric family satisfaction with care in the intensive care unit questionnaire, and the nurse professional competence scale-short version were used to collect data.

**Results:**

The findings of the study showed that the mean score of overall pFS-ICU was 80.0 ± 5.75 and the mean score of overall NPC-SV was 101.75 ± 19.66. Additionally, a significant strong positive correlation was reported between the overall NPC-SV and overall families’ satisfaction (*r* = 0.656, *p* < 0.001).

**Conclusion:**

Family satisfaction in the PICU is strongly influenced by nurses’ medical and technical competence and their ability to deliver well-organized, accurately documented care. While interpersonal, educational, and leadership competencies remain essential components of holistic nursing practice.

**Clinical trial number:**

Not applicable.

**Supplementary Information:**

The online version contains supplementary material available at 10.1186/s12912-026-04850-2.

## Introduction

The Pediatric Intensive Care Unit (PICU) is one of the most complex and stressful hospital environments, providing critically sick children with highly specialized and life-saving treatment. Advances in pediatric critical care have resulted in much higher survival rates; yet survival is no longer regarded as the only criterion of quality care. Increasing emphasis has been placed on family experiences and the competencies of healthcare workers, notably nurses, who play key roles in providing continuous, holistic care for critically ill children. Within this framework, family satisfaction and nursing care competencies have emerged as critical measures of quality in PICUs [1–3].

The application of Family-Centered Care (FCC), which prioritizes respect for family values, information sharing, parental participation, and collaboration, is strongly associated with family satisfaction in PICUs. According to recent reviews, FCC interventions like shared decision-making and parental participation in care rounds improve the overall care experience and parental satisfaction [3–5].

Hospitalized families in the PICU experience significant psychological stress, including anxiety, emotional exhaustion, and long-term disruptions. Emotional encouragement and reassurance from healthcare personnel have a major impact on parental satisfaction, emphasizing the necessity to address psychological requirements in high-quality pediatric treatment [2, 6, 7]. Nurses are the primary caregiver in PICUs, providing clinical and family assistance. Nursing care competencies include clinical knowledge, technological experience, rapid decision-making, and the ability to handle complex technologies. Communication, emotional support, and family engagement are all fundamental qualities that influence how family view care quality [6, 8].

Research shows a strong link between family satisfaction and nursing competencies. Family report higher levels of satisfaction when nurses are compassionate, accommodating, and communicate effectively. Nursing competencies have a direct impact on families’ impressions of the level of care provided in PICUs [6, 8, 9]. Family satisfaction is dependent on good communication between the nurse and the family. When children are unable to talk, families rely on nurses to provide regular updates, medical explanations, and emotional support. Nurse-led support therapies and structured communication approaches help to improve parental satisfaction, trust, and psychological well-being [6, 7, 9].

Even though family satisfaction and nurse competencies are recognized as quality indicators, little research has looked specifically at their relationship in PICUs. Organizational, training, and cultural factors all have an impact on family-centered approach implementation. More study is needed to determine the nursing competencies that have the greatest influence on family satisfaction [4, 5].

### Theoretical framework

FCC Model, FCC values partnerships between families and healthcare providers, emphasizing respect, information sharing, involvement, and collaboration as essential concepts. Implementing FCC in PICUs is linked to increased parental satisfaction and better family experiences [4, 5, 10]. Nursing competencies within the FCC, communication, emotional support, and family engagement are all necessary nursing skills for the FCC. Family expresses greater satisfaction when nurses display empathy, competence, and effective communication [6, 8, 9].

**Donabedian’s Structure**,** Process**,** and Outcome Model**, Donabedian’s model provides the theoretical structure for examining the relationship between nursing competencies and family outcomes. In this study, the structure component includes nurses’ demographic and professional characteristics, such as age, educational level, years of PICU experience, and previous training. The process component is represented by pediatric critical care nursing competencies as perceived by family members. The outcome component is represented by family satisfaction with nursing care in the PICU.

The integration of FCC and Donabedian’s model suggests that nurses’ structural characteristics influence their competencies, and that higher levels of nursing competencies, particularly in communication, emotional support, family engagement, and professional competence are associated with greater family satisfaction [4, 6]. The integrated framework, integrating FCC and Donabedian’s models demonstrates how nursing abilities enable family-centered practices, which result in higher family satisfaction [3–5].

Families in the PICU play an important role in the care of critically ill children. Family satisfaction is a significant predictor of care quality, influencing trust, adherence, and parental worry. The relationship between nursing competencies and satisfaction among families guides modifications to pediatric nursing education, training, and family-centered policies [1, 4, 5, 7].

### Aim of study

This study aimed to investigate the relationship between children’s family satisfaction and nursing care competencies in Pediatric Intensive Care Units.

### Specific objectives are

- Assess the nursing care competencies in Pediatric Intensive Care Units.
- Identify family satisfaction regarding care provided for their critically ill children in Pediatric Intensive Care Units.
- Investigate the relationship between children’s family satisfaction and nursing care competencies in Pediatric Intensive Care Units.

### Research hypotheses

- **H1**: There is a statistically significant positive relationship between nursing care competencies and family satisfaction with care in PICUs.
- **H0**: There is no statistically significant relationship between nursing care competencies and family satisfaction with care in PICUs.

### Operational definition of competency

In this study, competency is well-defined as the integrated application of nursing care, value-based nursing care, medical and technical care, pedagogy in nursing care, documentation and administration of nursing care, and development leadership and organization of nursing care through using validated self-reported score.

## Method

### Research design

A cross-sectional correlational research design was used to conduct this study.

### Settings

This study was conducted in five Pediatric Intensive Care Units at children’s hospitals related to different health areas in Alexandria as follows:

- **Alexandria University Hospitals** (El Shatby University Children’s Hospital, Smouha University Children’s Hospital).
- **General Authority for Health Insurance** (Sporting Students’ Hospital).
- **General Office of the Ministry of Health and Population** (El Raml Children’s Hospital, El Gomhoureya General Hospital).

### Participants

The study participants involved both pediatric critical care nurses and families of children who were admitted to PICUs. A convenient sample of 150 registered nurses in PICU out of 178 eligible nurses completed and returned the questionnaire (response rate, 92.7%) and provided direct bedside care for critically ill children. Their professional responsibilities incorporated child monitoring, medication administration, implementation of nursing interventions, documentation of care, and providing family-centered care. Regarding their educational background, nurses attached variable qualifications, including diploma in nursing, bachelor’s degree in nursing (BSN), and postgraduate nursing education. All participated nurses had prior clinical experience in critical care settings, with differing years of experience in PICUs.

**Fig. 1:**
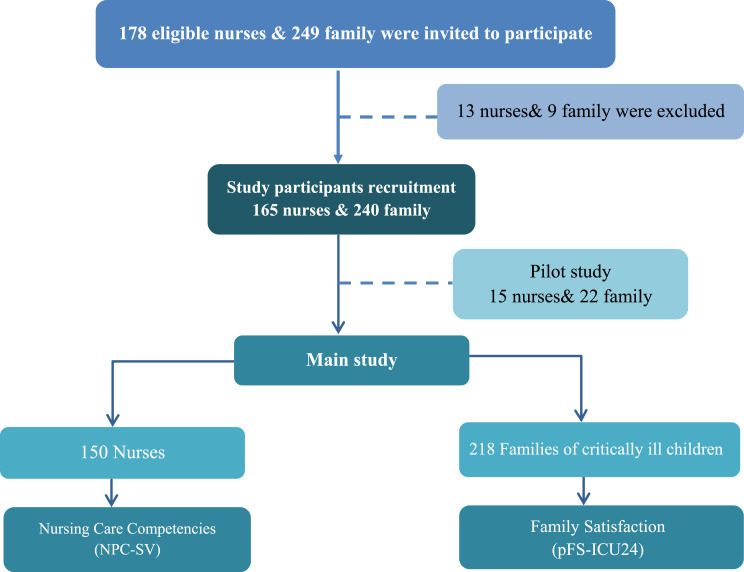
STROBE guidelines: the family satisfaction with nursing care competencies in pediatric intensive care units using pFS-ICU 24 and NPC-SV

Additionally, a convenient sample of 218 parent of critically ill children out of 249 eligible family members approached (response rate: 96.4%), who are admitted in the previously mentioned settings. Families of children who were admitted to the PICU within the child’s age ranged from one month to less than 18 years old, stayed for more than 48 h. in the PICU, and frequent visited or stayed at the bedside of their child for at least two days during the hospitalization in the PICU. Only one parent per family enrolled in the study. Children who died, transferred out, or discharged from the unit within 24 h. of admission were excluded from the study. This study was done in accordance with the Strengthening the Reporting of Observational Studies in Epidemiology (STROBE) Statement: guidelines for reporting observational studies [11] (Fig. 1).

The sample size study for children’s caregivers was estimated using the Epi info program version 7 (Centers for Disease Control and Prevention, Atlanta, USA) [12] with a population size of 362 children, an expected frequency of 50%, an acceptance error of 5%, and a confidence coefficient of 95%. The minimum sample size was calculated to be 187, and an additional 10% was included to account for dropouts.

### Ethical considerations

This study realized rigorous ethical principles to protect all participants, confirming compliance with international standards for medical research involving human subjects, directed by the World Medical Association’s latest reconsideration of the Declaration of Helsinki (October 2024) [13]. Approval from the Research Ethics Committee (REC) of the Faculty of Nursing, Alexandria University, was obtained before conducting the study on 15th June 2025 under serial number AU-20-4-367, with IRB 00013620 dated 19 September 2025. Written informed consent was obtained from the guardians of all children and pediatric critical care nurses before their enrollment in the study, after providing an appropriate explanation of the study’s purposes. The pediatric critical care nurses and families of critically ill children participate on voluntary basis and their right to withdraw from the study at any time was emphasized.

Data was collected by researchers who were not implicated in the direct clinical care of critically ill children and didn’t have any authority on treatment decisions. The individuals responsible for gathering data from the families were standalone from the healthcare team providing childcare to diminish any perceived pressure to participate. families were obviously informed that refusal to participate or withdrawal from study would not influence the quality of care delivered to their children in any way.

Correspondingly, data collected from pediatric critical care nurses were acquired by other researchers who were not engaged in nurses’ employment decisions, performance evaluation, promotion, hiring, or termination processes. The pediatric critical care nurses and families of critically ill children were secured that their participation was wholly voluntary and their right to withdraw from the study at any time was emphasized, and their responses would endure confidentiality and be used only for research purposes. To safeguard participants’ confidentiality, all gathered data were anonymized using coded identifiers, saved securely, and retrieved only by the research team.

### Data collection tools

Three tools were used to collect the data of the study.

#### Tool one: Children’s socio-demographic and clinical data record

This record was developed by the researchers to identify the child’s characteristics and clinical data and had two parts:

**Part I: Socio-demographic data of children** included child’s age, gender, level of education, birth order.

**Part II: Clinical data of children** involved diagnosis, medications, attached mechanical ventilation, and Pediatric Risk of Mortality (PRISM) score [14].

**The Pediatric Risk of Mortality** is a validated clinical severity scoring system used to approximate the risk of mortality in critically ill children. It is estimated based on physiological and laboratory variables gathered within the first 24 h of PICU admission. The total score ranges from 0 to 74, where higher scores indicate more physiological instability and predict higher risk of mortality, while lower scores indicate less severe illness and better physiological status.

#### Tool two: pediatric family satisfaction with care in the intensive care unit: pFS-ICU 24 questionnaire

Originally Heyland et al. (2002), previously developed and validated the Family Satisfaction in the Intensive Care Unit (FS-ICU 24) survey for adult family members of critically ill adults which consisted of 34 items [15]. While Wall et al. in 2007, refined and reduced it to a 24-item version, the FS-ICU 24 [16]. The pFS-ICU 24 is a psychometrically tested and extensively validated tool used to measure satisfaction with care and medical decision-making of parents/caregivers with children in the ICU (Cronbach’s alpha = 0.95) [17].

The pFS-ICU 24R is composed of two domains, satisfaction with care and satisfaction with medical decision-making. The survey contains a total of 24 Likert-scale questions, 14 questions designed to assess satisfaction with care, concern, ICU staff, waiting room, and ICU environment, and 10 questions designed to assess pediatric family satisfaction with decision-making regarding the care of critically ill patients and their information needs. In the questionnaire, 3 questions were related to the death of the child admitted to PICU (not applied in this study) and the last three questions in Part 2 were open-ended. The responses were recorded using a Likert scale 1–5 and then data were handled according to the pFS-24 questionnaire guidelines to get categories of very dissatisfied, slightly dissatisfied, mostly satisfied, very satisfied, and completely satisfied. The questionnaire was analyzed in three domains: satisfaction with care, satisfaction with decision-making, and overall satisfaction.

In addition, the questionnaire contains demographic data about the child’s family member and modified in this study and involved; gender, age, marital status, degree of kinship to the child, level of education of parents, number of siblings, previous admissions to PICU, and families perceived severity of illness of their child. The English version of the questionnaire was adopted in this study from website https://www.fsicu.ca/, to assess family satisfaction with the care given to the children and their family during the PICU stay. Additionally, pFS-ICU 24 in this study was translated into the Arabic version prior to for data collection to endorse clarity, comprehension, and cultural appropriateness for all participants.

### Translation and cultural adaptation of the translated arabic version of pFS-ICU 24

As no formally validated Arabic version of the pFS-ICU 24 questionnaire was found at the time of the study, a homogenous translation and cross-cultural adaptation process was operated before data collection. The translation process followed established guidelines for instrument adaptation. Initially, the original English version of the pFS-ICU 24 was independently translated into Arabic by two bilingual translators (translators 1 &2) fluent in both English and Arabic and familiar with medical terminology. Both translations were matched and produced into a single reconciled Arabic version. After that, the preliminary Arabic version was then back translated into English by two independent bilingual translators who were blinded to the original instrument (translator 3 &4). The back-translated versions were matched with the original questionnaire to ensure semantic and conceptual equivalence, and discrepancies were resolved through discussion among the research team.

### Validity and reliability of the translated arabic version of pFS-ICU 24

To ensure clarity and cultural suitability for the Egyptian context, the translated Arabic version of pFS-ICU 24 was tested for its content validity through reviewing committee in Pediatric Nursing Department, at Faculty of Nursing which included five professors in the department. Overall, responses for questionnaire significantly correlated and reflected high content validity index (CVI = 98.2). Cronbach’s alpha (α) was demonstrated to evaluate internal consistency reliability of questionnaire. Whereas the total scale demonstrated good internal consistency (α = 0.93. No modifications were made to both tools.

### Pilot study

A pilot study was carried out on 10% of family members of critically ill children (22 family members) to assess the clarity, the applicability of the translated Arabic version of pFS-ICU 24 and, estimated time for questionnaire filled. The pilot study confirmed that the questionnaire was clear, culturally appropriate, and easily understood by respondents, accordingly no modifications required. Subjects in the pilot study were excluded from the study.

#### Tool three: the nurse professional competence scale-short version (NPC-SV)

The original version of the Nurse Professional Competence (NPC) Scale was developed by Nilsson et al. (2014), which involves 88 items used to measure self-reported competence among registered nurses [18]. While in this study, the NPC- short version (NPC-SV) was used which reduced to 35 items by Nilsson et al. (2018) [19]. The NPC-SV assessed six competency domains which are nursing care (5 items); value-based nursing care (5 items); medical and technical care (6 items); pedagogy in nursing care (5 items); documentation and administration of nursing care (8 items) & development, leadership and organization of nursing care (6 items). Each item scored on a four-point Likert scale as follows;1 = a very low degree, 2 = a relatively low degree, 3 = a relatively high degree, 4 = a very high degree.

The total score ranged between 35 and 140. A higher score denoted higher nurse professional competence. In the current study, the Arabic version of the NPC-SV was adopted from Abuadas, (2023) [20]. Cronbach’s alpha values for the NPC-SV ranged from 0.71 to 0.86, indicating its acceptable reliability. Confirmatory factor analysis using principal components analysis, and the scale’s prior studies’ known-group validity all supported the scale’s construct validity [20].

Furthermore, data regarding socio-demographic characteristics of pediatric critical care nurses included age, gender, educational level, years of experience in PICU, hospital affiliation and marital status were attached to the tool.

### Data collection

The researcher interviewed every child’s parent individually in a special room in the PICUs to measure the pFS-ICU 24 after 48 h of their child admission to PICU in Arabic to permit initial adaptation to the critical care setting. The structured questionnaire taken 15–20 min to be filled. The nurse professional competence scale-short version with Arabic language was distributed on all pediatric critical care nurses inside the PICUs for all mentioned hospitals in the study, adequate time was allowed to fill out the questionnaire. It took roughly 20 and 30 min to complete the questionnaire. The data was collected over five months extending from beginning of July 2025 to the end of December 2025.

### Statistical analysis

Data was coded and analyzed using the Statistical Package for Social Sciences (SPSS), version 25. Descriptive Statistics were presented with frequency and percentage for categorical variables and mean ± standard deviation (SD) for continuous variables. Mean total scores and subscale scores of family satisfaction and nurse professional competence were calculated. The internal consistency reliability of tools was checked using Cronbach’s alpha coefficient(α). Shapiro-Wilk and Kolmogorov–Smirnov tests were applied to test the normal distribution of family satisfaction and nurse professional competence total scores. The Pearson correlation coefficient (r) was used to examine the relationship between normally distributed data. Correlation strength was interpreted as; less than 0.25 = weak, 0.26–0.50 = fair, 0.51–0.75 = moderate, and more than 0.75 = strong. Multiple linear regression analysis was conducted to identify the association between the nurses’ professional competences and family’s satisfaction regarding care in PICUs through the model. Assumptions of linear regression were tested before analysis. Regression analysis was used to investigate the strength and direction of association between nursing competence and family satisfaction while controlling related sociodemographic variables. The level of statistical significance was set at *p* < 0.05.

## Results

### Socio-demographic data of children’s parents

The study included 218 parents of critically ill children. More than half of children’s parents (59.2%) aged between 30 to less than 40 years old with mean age 33.9 ± 6.2 years. Additionally, it was found that the majority of children’s parents (88.5%) were females. In addition, it was reported that 55.5% of parents lived in rural areas. Moreover, 56.4% of parents had perceived moderate severity of illness of their children. Furthermore, 55% of parents had two siblings. Detailed information is presented in Table 1.

### Socio-demographic and clinical data of critically ill children in PICUs

Among the 218 critically ill children, slightly more than half of the children (50.9%) were aged less than one year, with a mean age of 32.8 ± 29.4 months, indicating a skewed age distribution with a higher proportion of infants alongside older children. Additionally, 61.5% of critically ill children were male and had second birth order. As result, more than half of children (62.8%) weren’t admitted to school. Moreover, it was found that more than half of critically ill children (53.2%) were admitted to intensive care units due to respiratory diseases. Nearly three quarters of the children (72.9%) had a history of previous PICU admission. Among those, 73.6% were admitted only once. In addition to that, antibiotics were prescribed for 55.5% of children. Only 15.6% of children were mechanically ventilated. The mean PRISM score for children was 8.1 ± 2.8, indicating a relatively low to moderate severity of illness among the studied critically ill children. Additional details are presented in Table 2.

### Families’ satisfaction with care in PICUs

The mean score of parents’ satisfaction with care was 49.0 ± 3.8. While the mean score of parents’ satisfaction with medical decision-making was 30.9 ± 4.7. Moreover, the mean score of overall pFS-ICU was 80.0 ± 5.7, indicating a generally high level of family satisfaction as shown in Table 3.

### Characteristics of pediatric critical care nurses in PICUs

A total of 150 pediatric critical care nurses participated in the study. Nearly half of the nurses (49.3%) aged from 30 to less than 40 years with mean age 33.9 ± 6.0 years. Additionally, it was found majority of nurses (83.3%) were female. In addition, more than one quarter of nurses (27.3%) had working experience in PICUs for more than 20 years with mean 11.9 ± 6.7 years. In relation to their educational level, 62.7% of nurses had bachelor’s degree in nursing. Further details are shown in Table 4.

### Professional competences of pediatric critical care nurses

The mean score of nurse’s professional competences regarding delivered nursing care and value-centered nursing care were 13.6 ± 3.7 and 15.8 ± 3.3, respectively. Additionally, the mean score of techno-medical care and pedagogy were 17.5 ± 3.2 and 14.0 ± 2.9, respectively. It was obvious from the table that nursing care documentation and administration registered the highest mean score, 24.0 ± 4.8. Moreover, the mean score of nursing care innovation, administration, and management was 16.7 ± 3.9. Consequently, the mean score of overall NPC-SV was 101.7 ± 19.6, as presented in Table 5.

### Correlation between parents’ satisfaction with care provided in the PICUs and the professional competences of pediatric critical nurses

It was obvious that there were significant moderate and positive correlations between parents’ satisfaction and all domains of nurses’ competencies in PICUs, including delivered nursing care, value-centered nursing care, techno-medical care, pedagogy in nursing care, nursing care documentation and administration, nursing care innovation, administration, and management (*r* = 0.544, 0.576, 0.622, 0.609, 0.625 & 0.522, respectively), *p* < 0.001. As result, a significant moderate positive correlation was reported between the Overall NPC-SV and overall families ‘satisfaction (r = 0.656, p < 0.001). Detailed correlation results are presented in Table 6.

### Multivariate linear regression shows the association between nursing professional competences and families’ satisfaction regarding care in PICUs

Regarding the NPC-SV domains, techno-medical care emerged as a significant positive association of pediatric family satisfaction (B = 0.532, β = 0.305, t = 2.958, *p* = 0.003), with a 95% confidence interval ranging from 0.177 to 0.886. Moreover, Nursing care documentation and administration showed a statistically significant positive association with pediatric family satisfaction (B = 0.407, β = 0.346, t = 2.366, *p* = 0.019), with a 95% confidence interval of 0.068 to 0.746. The multivariate regression analysis demonstrates that nurses’ professional competences have a measurable association with families’ satisfaction in PICUs. The overall NPC-SV score shows a strong positive and highly significant association with families’ satisfaction (B = 0.190, Beta = 0.656, CI = 0.161–0.200, *p* < 0.001), indicating that higher competence generally leads to greater satisfaction. The regression models are presented in Table 7.

**Table 1:** Socio- demographic data of critically Ill children’s parents in intensive care units (*n* = 218)

Parent’s data	No.	%
**Age (Years)**		
20-<30	48	22.0
30-<39	129	59.2
40 - <49	33	15.1
≥ 50	8	3.7
**Mean ± SD**	**33.96 ± 6.29**	
**Gender**		
Male	25	11.5
Female	193	88.5
**Residence**		
Rural	121	55.5
Urban	97	44.5
**Parents perceived severity of illness of their child**		
Mild	95	43.6
Moderate	123	56.4
Severe	0	0.0
**Number of siblings**		
No	24	11.0
1	19	8.7
2	120	55.0
3	55	25.2

**Table 2:** Socio-demographic and clinical data of critically Ill children in intensive care units (*n* = 218)

Children’s data	No.	%
**Child’s age**		
Less than 1 year (< 12 months)	111	50.9
From 1 to < 5 years (12–<60 months)	62	28.4
≥ 5 years (≥ 60 months)	45	20.6
Min-Max (months)	1–152	
Mean ± SD (months)	32.80 ± 29.43	
**Child’s gender**		
Male	134	61.5
Female	84	38.5
**Child’s birth order**		
First	43	19.7
Second	134	61.5
Third	41	18.8
**Child’s level of education**		
Didn’t enter school	137	62.8
KG	46	21.1
Primary	30	13.8
Other	5	2.3
**Diagnosis**		
Metabolic diseases (Diabetic Keto Acidosis)	22	10.1
Respiratory diseases (Pneumonia, Bronchitis)	116	53.2
Neurological diseases	25	11.5
Gastrointestinal tract diseases	55	25.2
**Previous admissions of child to PICU:**		
Yes	159	72.9
No	59	27.1
**Total number of child hospitalizations:***n* = 159		
1	117	73.6
2	19	11.9
3	23	14.5
**Prescribed medications ≠**		
Sedation	27	12.4
Anticonvulsant	27	12.4
Antibiotics	121	55.5
Insulin	22	10.1
Nebulizer therapy	50	22.9
Others (antiemetic, proton pump inhibitor, antifungal)	50	23.0
**Attached to mechanical ventilator**		
Yes	34	15.6
No	184	84.4
**PRISM score / Mean ± SD**	**8.10 ± 2.85**	

≠ more than one response

**Table 3:** Families’ satisfaction with care provided for their children in intensive care units (*n* = 218)

Families’ satisfaction	Mean	±SD
Satisfaction with care	49.01	3.87
Satisfaction with medical decision-making	30.99	4.72
**Overall** families**’ satisfaction**	**80.0**	**5.75**

**Table 4:** Characteristics of pediatric critical care nurses in intensive care units (*n* = 150)

Nurses’ characteristics	No.	%
**Age (Years)**		
20-<30	37	24.7
30-<39	74	49.3
≥ 40	39	26.0
Min-Max	22–44	
Mean ± SD	33.925 ± 6.077	
**Gender**		
Male	25	16.7
Female	125	83.3
**Marital status**		
Single	43	28.7
Married	107	71.3
**Years of experience in the Unit**		
< 5	27	18.0
5 - <10	34	22.7
10- <15	38	25.3
15 -<15	10	6.7
≥ 20	41	27.3
Min-Max	3–24	
Mean ± SD	11.933 ± 6.710	
**Education level**		
Technical Institute for Nursing	45	30.0
Bachelor’s degree in nursing	94	62.7
Postgraduate	3	2.0
Secondary School for Nursing	8	5.3

**Table 5:** The professional competences of pediatric critical care nurses (*n* = 150)

The nurses’ professional competences	Mean	±SD
Nursing care	13.65	3.75
Value-based nursing care	15.81	3.38
Medical and technical care	17.50	3.26
Pedagogy in nursing care	14.03	2.90
Documentation and administration of nursing care	24.06	4.88
Development leadership and organization of nursing care	16.71	3.95
**The overall NPC-SV**	**101.75**	**19.66**

**Table 6:** Correlation between families’ satisfaction regarding care provided and professional competence of pediatric critical care nurses

Nurses’ professional competences		Satisfaction with care	Satisfaction with medical decision-making	Overall pFS-ICU 24
Nursing care	**r**	0.303*	0.414*	0.544*
	**p**	< 0.001*	< 0.001*	< 0.001*
Value-based nursing care	**r**	0.358*	0.408*	0.576*
	**p**	< 0.001*	< 0.001*	< 0.001*
Medical and technical care	**r**	0.319*	0.496*	0.622*
	**p**	< 0.001*	< 0.001*	< 0.001*
Pedagogy in nursing care	**r**	0.390*	0.421*	0.609*
	**p**	< 0.001*	< 0.001*	< 0.001*
Documentation and administration of nursing care	**r**	0.394*	0.438*	0.625*
	**p**	< 0.001*	< 0.001*	< 0.001*
Development leadership and organization of nursing care	**r**	0.334*	0.362*	0.522*
	**p**	< 0.001*	< 0.001*	< 0.001*
**Overall NPC-SV**	**r**	0.395*	0.474*	0.656*
	**p**	< 0.001*	< 0.001*	< 0.001*

r: Pearson coefficient *: Statistically significant at p ≤ 0.05

**Table 7:** Multivariate linear regression showing the associations between the nurse professional competences and families’ satisfaction regarding care in PICUs

Nurse professional competence	B	Beta	t	*p*	95% CI	
					LL	UL
Nursing care	0.303	0.199	1.919	0.056	-0.008	0.614
Value-based nursing care	0.149	0.087	0.801	0.424	-0.218	0.517
Medical and technical care	0.532	0.305	2.958*	0.003*	0.177	0.886
Pedagogy in nursing care	0.538	0.275	1.562	0.120	-1.217	0.141
Documentation and administration of nursing care	0.407	0.346	2.366*	0.019*	0.068	0.746
Development leadership and organization of nursing care	0.146	0.103	1.136	0.257	-0.107	0.399
**R**^**2**^**=0.451**,** adjusted R**^**2**^ **= 0.435**, F = 28.841*,***p*** < 0.001*						
**Overall NPC-SV**	0.190	0.656	12.762*	< 0.001*	0.161	0.200
**R**^**2**^**=0.430**,** adjusted R**^**2**^ **= 0.427**, F = 162.869*,***p*** < 0.001*						

F, p: f and p values for the model R2: Coefficient of determination B: Unstandardized Coefficients

Beta: Standardized Coefficients t: t-test of significance CI: Confidence interval

LL: Lower limit UL: Upper Limit *: Statistically significant at *p* ≤ 0.05

## Discussion

### Overall family satisfaction with care in the PICU

The present study demonstrated a high level of overall family satisfaction with care and medical decision-making in the PICU, with a mean overall satisfaction score of 80.0 ± 5.75. This finding suggests that families perceived the care provided as effective, safe, and professionally delivered. Such high satisfaction may be associated with strong nursing performance across several professional competence domains, particularly coordination of care, emotional support, and effective communication. In the PICU environment, where families experience intense emotional distress and uncertainty, visible clinical competence and clear information provision are critical determinants of perceived care quality.

The high level of family satisfaction observed in the present study should also be interpreted cautiously. Satisfaction measures in healthcare research may be influenced by potential ceiling effects, where respondents tend to report high scores. In addition, cultural factors may influence parents’ responses, as families in some settings may hesitate to express dissatisfaction with healthcare professionals. Furthermore, the timing of data collection during hospitalization may have contributed to higher satisfaction ratings, as families were still receiving care for their children.

These findings are consistent with those reported by Cintra et al. (2022), who identified high levels of family satisfaction in Brazilian PICUs, particularly in the domains of professional attitude, care and cure, information provision, and family participation [21]. Similarly, Sahota et al. (2025), reported that most parents in a tertiary PICU in Northern India were satisfied with the care provided, emphasizing the role of staff competence and communication [22]. In a European context, Terp et al. (2025), also reported generally positive family evaluations of PICU care, particularly regarding trust in healthcare professionals [10]. Together, these studies suggest that family satisfaction in PICUs is consistently associated with nurses’ professional competence and effective communication across diverse healthcare settings.

In contrast, Zaitoun (2024), reported lower levels of selected nursing competence domains, particularly leadership and professional development, among nurses in Palestine [23]. These differences may reflect variation in healthcare infrastructure, workload, and access to continuing education. Importantly, Zaitoun (2024), demonstrated that participation in training workshops significantly improved nurses’ competence, supporting the interpretation that the high satisfaction observed in the present study may be linked to institutional support for professional development [23].

### Relationship between nurse professional competence and family satisfaction

A statistically significant and strong positive correlation was identified between overall nurse professional competence and family satisfaction with care in the PICU (*r* = 0.656, *p* < 0.001). This finding indicates that families of critically ill children tend to evaluate care quality in relation to observable nursing competencies, including technical proficiency, clinical judgment, and the ability to manage complex clinical situations safely and efficiently.

This result aligns with the findings of Munir et al. (2025), who reported high family satisfaction with nursing care in a pediatric hospital, particularly in relation to professionalism and communication [24]. Similarly, Aiken et al. (2018) demonstrated that higher levels of nursing competence were associated with improved patient satisfaction outcomes [25]. Collectively, these findings reinforce the central role of nursing professional competence as a key determinant of family satisfaction in acute and critical care settings.

### Predictors of family satisfaction: medical, technical, and administrative nursing care

Multivariate regression analysis revealed that medical and technical nursing care, as well as documentation and administration of nursing care, were significant predictors of family satisfaction. This finding may suggest that families place particular value on nurses’ technical competence and their ability to deliver well-organized and accurately documented care. In the PICU, where patient acuity is high and clinical outcomes are uncertain, such competencies may provide families with reassurance regarding patient safety and continuity of care.

These findings are supported by Johnson et al. (2016), who demonstrated that accurate nursing documentation enhances continuity of care, reduces errors, and improves communication among healthcare providers [26]. Likewise, Müller et al. (2018), emphasized that structured communication and documentation practices contribute to improved patient safety and care coordination [27]. From the family perspective, efficient documentation and administrative organization may enhance trust in the healthcare team and overall satisfaction with care.

Conversely, previous studies have highlighted the importance of interpersonal and emotional aspects of care. Eltaybani and Ahmed (2021), reported that communication, emotional support, and family involvement in decision-making were significant contributors to family satisfaction in intensive care units [28]. Hellín et al. (2022), similarly identified nurse–family communication and interpersonal care as key determinants of satisfaction [29]. The divergence between these findings and the current results may be explained by the pediatric critical care context, in which families may prioritize technical competence and accurate care management due to heightened concerns about safety and survival.

### Cultural and contextual considerations

The interpretation of these findings should be considered within the cultural and contextual framework of the study setting. In family-centered and collectivist societies, families of critically ill children may prioritize observable clinical competence and structured care processes over less visible aspects of nursing practice, such as leadership or educational roles. Additionally, the emotional burden associated with having a child admitted to the PICU may intensify families’ focus on technical proficiency and documentation accuracy, which are directly associated with perceptions of safety and quality of care.

### Non-significant predictors: value-based care, education, and leadership

Although value-based nursing care, nursing education, and leadership and organizational competencies demonstrated positive correlations with family satisfaction, they did not remain significant predictors in the multivariate model. These competencies may be less directly observable to families compared with technical and administrative nursing activities. Leadership and educational roles often operate at a systems level, which may limit families’ awareness of their impact on daily care delivery.

This finding partially contrasts with previous literature emphasizing the importance of ethical care, family involvement, and communication in intensive care settings. Al-Mutair et al. (2013) highlighted that addressing family needs and promoting involvement in care and decision-making are essential components of satisfaction [30]. However, in pediatric critical care contexts, concerns regarding patient safety and clinical outcomes may outweigh the influence of less visible nursing competencies.

The borderline non-significant effect of general nursing care supports the conceptualization of nursing competence as a multidimensional construct. Nilsson et al. (2014) emphasized that the relative influence of competence domains varies according to the clinical context and the outcome being examined [18]. In the PICU setting, advanced technical and administrative competencies may therefore have greater explanatory power in predicting family satisfaction.

### Clinical implications for pediatric nursing in critical care settings

The findings of this study have important implications for nursing practice in critical care settings. Professional development initiatives in PICUs should prioritize strengthening nurses’ medical and technical competencies, as well as documentation and administrative skills. Incorporating structured competence assessment tools into routine performance evaluations may support ongoing monitoring and enhancement of nursing practice. Furthermore, reinforcing high-quality documentation practices may improve continuity of care, interdisciplinary communication, and family trust in critical care services.

Several methodological considerations should be acknowledged when interpreting these findings. Potential response bias may have occurred because family satisfaction was measured using self-reported questionnaires. In addition, unmeasured confounding variables, such as severity of illness or previous hospitalization experiences, may have influenced parents’ perceptions of care. Finally, because the study was conducted in specific PICU settings, the generalizability of the findings to other healthcare contexts should be interpreted with caution.

## Conclusion

In conclusion, family satisfaction in the PICU is strongly influenced by nurses’ medical and technical competence and their ability to deliver well-organized, accurately documented care. These findings underscore the critical role of professional nursing competence in shaping family experiences in pediatric critical care. While interpersonal, educational, and leadership competencies remain essential components of holistic nursing practice, further research is needed to explore strategies for enhancing their visibility and perceived value to families in PICU settings.

### Study limitations

Despite the strength of the findings, family satisfaction represents a subjective evaluation of care and may not fully capture all dimensions of nurses’ professional competence. Nurses’ competencies were rated using self-reported measures only, which may introduce response bias, involving the risk of overestimating their own skills. No objective measures, such as direct observational assessments, or child outcome indicators, were operated to authorize the reported competencies. Hence, the findings should be illuminated with caution, and future studies are recommended to incorporate more objective methods of competency assessment to reinforce the validity of the results. Families’ perceptions may be influenced by emotional stress, cultural expectations, and the acute nature of the PICU environment. Families of children who had died in PICU were excluded from the study owing to ethical considerations and to evade causing additional emotional distress during an extremely sensitive phase of bereavement. Therefore, future studies specifically explore the perspectives of bereaved families to gain deeper insight into end-of-life care experiences in the PICU. Future research combining family satisfaction measures with objective assessments of nursing competence may provide a more comprehensive understanding of factors influencing family satisfaction. The study incorporated nurses from five different intensive care units, where variations in unit culture, clinical experience, and populations may have affected the findings. Therefore, the findings should be illuminated with consideration of potential unit-specific variability. A convenience sampling technique was applied to recruit participants, which may advance selection bias and boundary the representativeness of the sample.

## Supplementary Information

Below is the link to the electronic supplementary material.

Supplementary Material 1

## Data Availability

The datasets generated during the current study are available from the corresponding author on reasonable request.
