# Supplementary material for: From crisis to care: evaluating nursing competencies in pediatric intensive care units through family eyes
Source: BMC Nurs. 2026 Jun 16;25:544. doi: 10.1186/s12912-026-04850-2 (PMC13270677; doi:10.1186/s12912-026-04850-2)
Supplement: Supplementary file 1 — Supplementary Material 1 [file 12912_2026_4850_MOESM1_ESM.docx]

**Table (1): The Parent’s Suggestions Regarding Improvements in ICUs Services (n= 218)**

| **Open ended items** | **No.** | **%** |
| --- | --- | --- |
| **Q28**: **Do you have any suggestions on how to make care provided in the ICU better?** | | |
| - No | 152 | 69.7 |
| - Involve child’s caregiver in child’s care | 38 | 17.4 |
| - Increase number of PICU staff | 28 | 12.8 |
| **Q29: Do you have any comments on things we did well?** | | |
| - Yes | 0 | 0.0 |
| - No | 218 | 100.0 |
| **Q30: Do you have any suggestions on how we could improve the ICU experience for you and your child?** | | |
| - No | 115 | 52.8 |
| - Increase duration of visiting in PICU | 42 | 19.3 |
| - Make all medication available | 34 | 15.6 |
| - Availability for clinical radiography | 27 | 12.4 |
